# Impact of Workplace on the Risk of Severe COVID-19

**DOI:** 10.3389/fpubh.2021.731239

**Published:** 2022-01-05

**Authors:** Tsuyoshi Nakamura, Hiroyuki Mori, Todd Saunders, Hiroaki Chishaki, Yoshiaki Nose

**Affiliations:** ^1^Faculty of Environmental Science, Nagasaki University, Nagasaki, Japan; ^2^Department of Life Creation, Nagasaki Women's College, Nagasaki, Japan; ^3^Graduate School of Biomedical Science, Nagasaki University, Nagasaki, Japan; ^4^Department of Family Practice, National Health Insurance Clinic, Nakatsu, Oita, Japan; ^5^Graduate School of Medical Sciences, Kyushu University, Fukuoka, Japan

**Keywords:** COVID-19, severe disease, workplace, occupation, cohort study, relative risk, lockdown, direct customer exposure

## Abstract

Indiscriminate regional lockdowns aim to prevent the coronavirus disease 2019 (COVID-19) infection by restricting the movement of people; however, this comes with psychological, social, and economic costs. Measures are needed that complement lockdowns and reduce adverse effects. Epidemiological studies, to date, have identified high-risk populations, but not workplaces appropriate for closure. This study was conducted to provide evidence-based measures that used exact and reliable follow-up data of the PCR-positive COVID-19 cases to complement lockdowns. The data are not subjected to selection or follow-up biases, since the Japanese government, by law, must register and follow all the PCR-positive cases until either recovery or death. Direct customer exposure may affect the quantity of viral inoculum received, which, in turn, may affect the risk of the severity of disease at infection. Therefore, the professions of the cases were grouped according to their frequency of direct customer exposure (FDCE) based on subjective observations, which resulted in five workplaces; hospital, school, food service, outdoor service, and indoor office being identified. Analyzing the follow-up data, we obtained precise estimates for the risk of severe disease, defined as intensive care unit (ICU) hospitalization or death, for the workplaces adjusted for age, sex, family status, and comorbidity. Major findings are as follows: hospital and school are the lowest risk, food and outdoor services are, despite higher FDCE, safer than indoor office. Unemployed and unclear are the highest risk, despite low FDCE. These results suggest the following workplace-specific measures complementing the lockdown: school should not be closed and indiscriminate closing of food and outdoor service industries should be avoided, since it would be more effective to reinforce their efforts to promote adherence to public health guidelines among students and customers. These actions would also reduce the adverse effects of the lockdown. This study is the first to address the causality between the workplaces and severe disease. We introduce FDCE and adherence to public health guidelines (APHGs) to associate the workplace characteristics with the risk of COVID-19 severity, which provided the basis for the measures complementing lockdowns.

## Introduction

The novel coronavirus disease 2019 (COVID-19), first appeared in Wuhan, China at the end of 2019, spread globally and as of March 11, 2021 has resulted in 2,624,677 deaths worldwide (1). In addition to this loss of life, measures to control the spread of the disease have resulted in the severe economic consequences for citizens and countries around the world (2).

Different efforts of the countries to control its spread include a variety of management techniques such as travel bans, quarantines, lockdowns, and mask mandates (3). Versions of these include restrictions, both total and partial on those entering countries from the high COVID-19 rate countries, domestic quarantines for arriving individuals, total and partial lockdowns based on the current situation at the moment, and federal, state, city, town, and private business mask rules (3–11). By April 2020, more than 90 countries were under various forms of lockdowns, resulting in about half of the population of world having been asked or ordered to stay at home by their governments (4). Nevertheless, the pandemic had not abated and the prevalence and mortality due to the COVID-19 in 27 countries failed to show a significant decline 15 days after the lockdown compared to the 15 days before the lockdown (2).

Although lockdowns have, despite other consequences, been effective in reducing the COVID-19 cases in many countries and/or regions during periods of rapid spread of the virus, they have caused mental health issues (5, 6), disrupted social lives (7, 8), decreased access to food and healthcare (9), and resulted in business closures and loss of employment and income (10). School closures have led to an unprecedented negative impact on education (11) with over 200 million students enrolled at primary, secondary, and higher levels of education being affected as of February, 2021 (12). In Japan, as a result of the suspension of operations in much of the service industry, many workers lost their jobs. There has also been an important psychological toll with 6,976 women in Japan taking their lives last year, a nearly 15% increase from 2019 which was the first year-over-year increase in more than a decade (13). While social distancing measures have decreased the transmission rate, they have failed to decrease the number of cases infected with the COVID-19 (14).

Since global deaths from the COVID-19 continue to increase, world governments and agencies are still working toward understanding who is most at risk of death (15). In Sweden, income and education levels were significantly associated with the COVID-19-related deaths (16), while in England, deprivation (essentially a measure of poverty) was positively associated with the COVID-19-related deaths (17). The risk of death for essential workers and healthcare workers (HCWs) has been well documented (18–20). In Britain, the risk of the COVID-19 deaths or intensive care unit (ICU) hospitalization for HCW is estimated to be 7.4 times that of non-essential workers (21). However, since the agreement between job at baseline 2006–2010 and the follow-up period 2014–2019 for a subsample of the cohort was substantially lower, 67% to 92%, their risk estimates would be subjected to biases due to the misclassifications (22).

While these previous findings can help to determine high-risk groups in need of administrative assistance, they are not immediately tied to any effective public health measures reducing the risk of severity of the COVID-19. More detailed results and some insight into the causality between the disease severity and biosocial factors are needed to generalize the results to find the cost-effective preventive measures.

The viral pathogenesis theory states that the severity of disease is proportionate to the viral inoculum received (23) and Gandhi and Rutherford (23) argued that universal masking could become a form of “variolation” that would generate immunity thereby helping reduce the severity of disease and ensuring that a greater proportion of new infections are asymptomatic. The quantity of viral inoculum received is also possibly affected by such measures as the disinfecting of hands and adequate ventilation. Importantly, contact frequency (CF) with others inside of the minimum centers for disease control and prevention (CDC) recommended social distance of 6 feet (24) and the degree of adherence to public health guidelines (APHGs), which the CDC defines as wearing a mask that covers the mouth and nose, staying at least 6 feet apart, washing the hands often, and getting vaccinated where possible (25), may affect the quantity of viral inoculum received, which, in turn, may affect the risk of the severity of disease at infection. Lockdowns that restrict the movement of people or business operations reduce CF, but do not necessarily promote APHG.

Research with the objective of estimating the occupational risk related to the COVID-19 severity is rare and more data are needed to further elucidate the occupation-specific risks to ensure workplace safety (26). Since the COVID-19 is designated a type II infectious disease, Japanese public health centers, by law, must register all the PCR-positive cases and follow them until recovery or death without exception. This follow-up data comprises an ideal cohort with no selection and no follow-up biases. The objectives of this study are to precisely estimate the risk of the severity of the COVID-19 intrinsic to workplaces by using these ideal cohort data and address workplace-specific interventions complementing the lockdowns.

## Materials and Methods

### Subjects

Doctors identifying the COVID-19 PCR test positive cases must fill out a “Notification of Outbreak” form, specified by the Japanese Ministry of Health, Labor and Welfare, and send it to a city public health center. These centers register patient information to share with the government office and the other public health centers, assign patients to home or hotel quarantine, or hospitals, and track them until either recovery or death. With the exception of personal identification such as name and address, these centers publicize all the case information on the internet. For this study, we downloaded this case information for all the patients, regardless of age, registered in Osaka prefecture, population 8.8 million, between February 20th and September 15th, 2020. The data comprise ideal cohort data with no selection and no follow-up biases. Excluding 14 cases whose PCR samples were collected after death, we have 9,690 COVID-19 cases for analysis. Ten cases of those who died of other diseases while hospitalized for COVID-19 treatment were categorized as the COVID-19 deaths, since the COVID-19 was considered to have aggravated the diseases. Provided material in the Supplement, it describes the method for access to the original data and transformation to an EXCEL file.

### Outcome

In the COVID-19 follow-up reports, disease severity is classified as asymptomatic and symptomatic but mild or ICU hospitalized and cases are either recovered or deceased at discharge. Our outcome of interest is “the severe COVID-19” defined as either died or hospitalized in the ICU. The proportion of severe cases will be termed as severe rate.

### Workplace

Since the risk of the COVID-19 infection increases with the entry of others inside a critical social distance (24), the risk of infection should be different among workplaces where the frequency of direct customer exposure (FDCE) is different. Since it is difficult to obtain a representative value of FDCE for each workplace, we will consider the difference in FDCE between workplaces according to the characteristics of each workplace specialty. We classified the professions of the COVID-19 cases in consideration of FDCE into the following five workplace categories: hospital, school, indoor office (private companies and government offices), food service (restaurants, bars, and supermarkets), and outdoor services (e.g., construction, transportation, and security). Outdoor service is comprised of a profession whose work is conducted mostly outside or interactively with unspecified customers.

Hospitals engulf a greater risk than schools due to higher exposure to symptomatic people with the COVID-19 and due to increased density of exposure. However, hospitals and schools have the following features in common. Hospital and school staff often interact with patients and students inside a critical social distance, respectively. In hospitals, healthcare professionals such as doctors and nurses set an example for observing health regulations to prevent nosocomial infections and hospital staff and patients have regular physically close contact. Schools are similar to hospitals in that faculty and staff set an example for observing health regulations to prevent interstudent transmission and students follow this example. FDCE for food service and outdoor service is relatively higher compared to indoor office.

Table 1 shows the professions, described in the Notification of Outbreak form, grouped by workplace and the frequency of cases. Unemployed in Table 1 includes housewives, pensioners, and those looking for jobs, while unclear consists of those who declined or failed to identify their professions. Unemployed individuals have virtually no customers and, therefore, a relatively lower FDCE than indoor office individuals. FDCE in the unclear workplace is not known because their professions are not clearly specified. FDCE assigned to the workplaces is summarized in Table 1. While FDCE may differ according to country, the data here references Japan.

**Table 1:** Professions classified according to workplace.

**Workplace**	**Profession**	**Freq**.	**Total**	**FDCE**
Hospital	Medical staff	539		High
	Nursing care staff	168		
	Elderly care staff	49		
	Hospital chef	22		
	Welfare staff	7		
	Nutritionist	1	786	
School	Student	838		High
	Teacher	153		
	Monk	1	992	
Indoor Office	Private company employee	2,036		Medium
	Government employee	145	2,181	
Food Service	Restaurant and bar employee	1,250		High
	Supermarket employee	4	1,254	
Outdoor Service	Self-employed	539		High
	Day Laborer	180		
	Construction worker	113		
	Retail sales	87		
	Salesperson	76		
	Factory worker	49		
	Driver	33		
	Transporter	25		
	Delivery	17		
	Real estate employee	13		
	Security guard	13		
	Instructor	12		
	Cleaning	10		
	Demolition	8		
	Painter	5		
	Home helper	3		
	Contractor	3		
	Advertising	1		
	Welding	1	1,188	
Unemployed	Unemployed	1,730	1,730	Low
Unclear	No description	1,559	1,559	Unknown

### Stage 0 and 1

**Figure 1:**
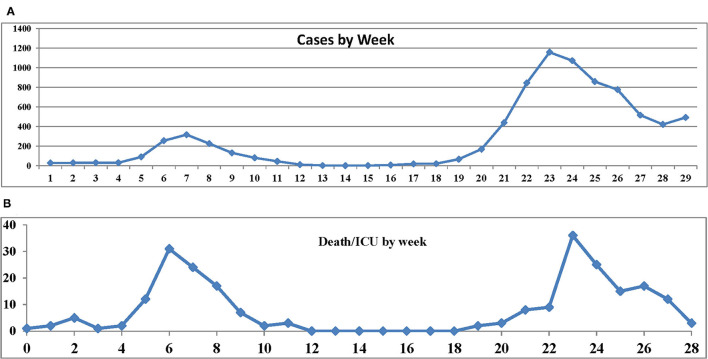
**(A)** Number of the coronavirus disease 2019 (COVID-19) cases. **(B)** Number of severe cases by week since February 20th.

Figure 1 shows the number of the COVID-19 cases (A) and severe cases (B) by week since February 20th, 2020. Table 2 shows the date of the first day of each week. The first wave ended at week 12, but began to spread again at week 18. Weeks 0–15 will be denoted by stage 0 and weeks 16–28 by stage 1.

**Table 2:** Date of the first day of each week.

**Week**	**0**	**1**	**2**	**3**	**4**	**5**	**6**	**7**	**8**	**9**
**1st Day**	2/20	2/27	3/5	3/12	3/19	3/26	4/2	4/9	4/16	4/23
**Week**	**10**	**11**	**12**	**13**	**14**	**15**	**16**	**17**	**18**	**19**
**1st Day**	4/30	5/7	5/14	5/21	5/28	6/4	6/11	6/18	6/25	7/2
**Week**	**20**	**21**	**22**	**23**	**24**	**25**	**26**	**27**	**28**	**29**
**1st Day**	7/9	7/16	7/23	7/30	8/6	8/13	8/20	8/27	9/3	9/10

The objective of this study is to assess the impact of workplace on the severity of disease adjusted for background biosocial factors such as age, sex, family (family members living together), and comorbidity. Table 3 presents the levels and codes of the variables. The frequencies of cases, the number of severe cases, and the proportion of severe cases (rate) are shown by stage. Hereafter, the rate will be referred to as severe rate. Since professions in hospital and school categories rarely developed severe disease, these were combined to create a level termed as hospital/school food and outdoor services were combined to create a level termed as service.

**Table 3:** Frequency of severe cases and severe rate (%) by stage.

		**Stage 0**			**Stage 1**		
**Variable**	**Code: level**	**Freq**.	**Severe**	**Rate (%)**	**Freq**.	**Severe**	**Rate (%)**
Age	20: 0–29	351	0	0	3,460	0	0
	40: 30–49	511	8	1.57	2,252	1	0.04
	60: 50–69	364	30	8.24	1,445	31	2.15
	80: 70–99	268	78	29.1	1,039	106	10.2
Sex	0: Female	674	41	6.08	3,555	42	1.18
	1: Male	820	75	9.15	4,639	96	2.07
Family status	0: With	879	51	5.8	5,228	56	1.07
	1: No	249	19	7.63	2,427	58	2.39
	2: Unclear	366	46	12.57	541	24	4.44
Workplace	0: Hosp./Sch.	240	2	0.833	1,538	1	0.065
	1: Service	235	11	4.681	2,207	9	0.408
	2: Indoor Office	343	15	4.373	1,838	7	0.381
	3: Unemployed	210	30	14.29	1,520	86	5.66
	4: Unclear	466	58	12.45	1,093	35	3.2
Comorbidity	0: No	1,329	83	6.25	7,387	81	1.1
	1: With	165	33	20	809	57	7.05
D/I	0: No	1,378		0.92	8,058		0.98
	1: Yes	116		0.08	138		0.02

### Statistical Analysis

First, we define the endpoint D/I as D/I = 1 for severe cases and D/I = 0 for otherwise cases. We also define the binary variable stage as stage = 0 for cases in stage 0 and stage = 1 for cases in stage 1. Then, we define age_50 = Max (age-50, 0). In other words, age_50 = 0 while age ≤ 50 and age_50 = age-50 for age > 50. This is a piecewise linear function with one change point at age = 50. Age_60 and age_70 are similarly defined. These functions fit the data better than age only with the least loss of efficiency (27).

Since family has three levels 0, 1, and 2, we define one piecewise linear function family_1 = Max (family-1, 0). That is, family_1 = 0 if family = 0 or 1 and family_1 = 1 if family_2 = 2. The binary variables such as sex, comorbidity, and stage are used. An interaction term age_60 × comorbidity, defined as a multiplication of two variables age_60 and comorbidity, is used to deal with age-dependent effect of comorbidity.

For workplace, we define an indication function Job * k for each level k as follows: Job * k = 1 if level = k and Job * k = 0 otherwise (k = 1, 2, 3, 4). It follows that Job * 1 = Job * 2 = Job * 3 = Job * 4 = 0 if and only if level = 0.

We examined associations between D/I and workplace, age, sex, family, and comorbidity by using a logistic model. It is disadvantageous that the logistic model presents the odds ratio (OR) but not the relative risk (RR), since RR is straightforward to interpret. Therefore, we apply a modified logistic model (28) to obtain a precise estimate of RR as follows: First, an ordinary stepwise logistic model is applied to select significant variables and then a modified logistic model by using the selected variables is applied. The results present precise estimates of RR for the levels of each variable, but do not provide their SDs.

To investigate relationships between the severe rates in 1st stage and 2nd stage, a simple linear regression model *y* = α + βx + ε, ε ~ N (0, σ^2^) was applied, where α is a constant, β is a regression coefficient, and *x* and *y* take values log (severe rate) in stage 0 and stage 1, respectively.

## Results

### Severe Rates by Stage

Table 4A shows the frequencies of cases and severe cases and severe rates by workplace and stage. Table 4B shows the frequency of cases and severe cases, odds, OR, risk, and RR for the pooled data.

**Table 4:** Frequency of cases and severe cases by workplace: (A) By stage. (B) Pooled.

**(A)**						
**Workplace**	**Stage 0**			**Stage 1**		
	**Freq**.	**Severe**	**Rate (%)**	**Freq**.	**Severe**	**Rate (%)**
Hospital	157	1	0.64	629	1	0.16
School	83	1	1.2	909	0	0
Food	97	3	3.09	1,157	2	0.17
Outdoor	138	8	5.8	1,050	7	0.67
Company	313	13	4.15	1,723	7	0.41
Government	30	2	6.67	115	0	0
Unemployed	210	30	14.29	1,520	86	5.66
Unclear	466	58	12.45	1,093	35	3.2
**(B)**						
**Workplace**	**Pooled**					
	**Freq**.	**Severe**	**Odds**	**OR**	**Risk**	**RR**
Hospital	786	2	0.0026	1	0.0025	1
School	992	1	0.001	0.4	0.001	0.4
Food	1,254	5	0.004	1.57	0.004	1.6
Outdoor	1,188	15	0.0128	5.01	0.0126	5.04
Company	2,036	20	0.0099	3.89	0.0098	3.92
Government	145	2	0.014	5.48	0.0138	5.52
Unemployed	1,730	116	0.0719	28.2	0.0671	26.8
Unclear	1,559	93	0.0634	24.9	0.0597	23.9

**Figure 2:**
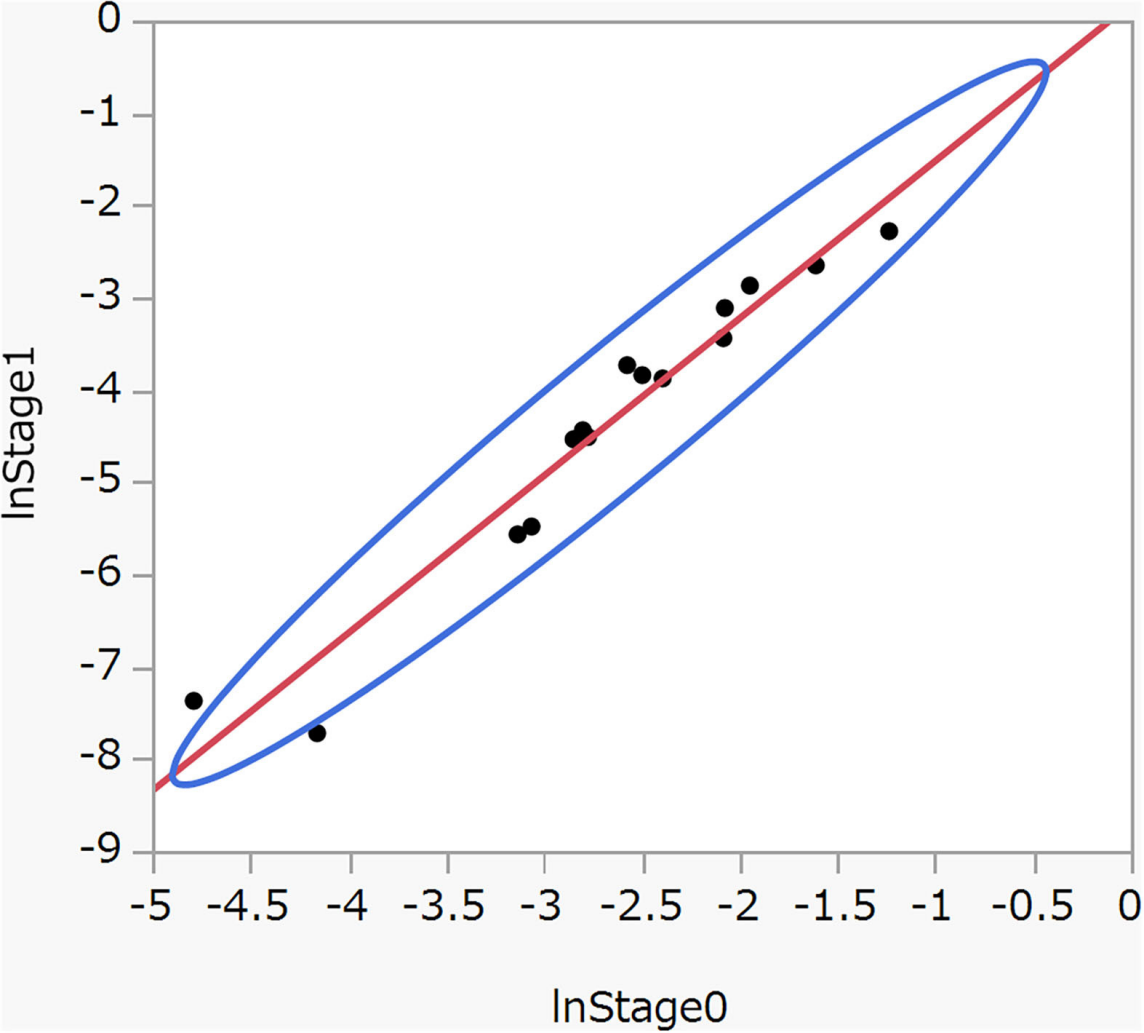
Scatter plot between log (severe rate) in stage 0 and stage 1 with 95% density ellipse.

Figure 2 shows a scatter plot between log (severe rate) in stage 0 and stage 1 in Table 4A. Applying a linear regression model *y* = α + βx + ε, we have 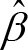 = 1·705 (SE = 0.114, *p* < 0·001), 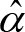 = 0·18 (SE = 0.321, *p* = 0.59), and *R*^2^, the coefficient of determination adjusted for the degree of freedom, is 0.94. The test for the normality of residuals was 0.208 (*p* = 0.84). The results suggest it approximately holds that *y* = 1.7*x* + ε. Briefly, severe rates in stage 1 are approximately determined by corresponding severe rates in stage 0 plus random errors, irrespective of the levels of the variables. These finding prompted us to pool stage 0 and stage 1 in estimating the risk of severe disease associated with the levels of the variables.

### Naïve OR and RR

Table 5 shows the frequency of severe cases, OR, and RR. RR for age 70–99 years compared to age 30–49 years is extremely high: about 43. RR of unemployed and unclear compared to hospital/school is also remarkably high: about 41 and 36, respectively.

**Table 5:** Frequency of severe cases and odds ratio (OR) and relative risk (RR).

**Variable**	**Code**	**Level**	**Severe**	**Freq**.	**Odds**	**Risk**	**OR**	**RR**
Age	20	0–29	0	3,811	0	0	0	0
	40	30–49	9	2,763	0.0033	0.003	1	1
	60	50–69	61	1,809	0.0349	0.034	10.7	10.4
	80	70–99	184	1,307	0.1,638	0.141	50.1	43.2
Sex	0	Female	83	4,229	0.02	0.02	1	1
	1	Male	171	5,459	0.0323	0.031	1.62	1.6
Family	0	With	107	6,107	0.0178	0.018	1	1
	1	No	77	2,676	0.0296	0.029	1.66	1.64
	2	Unclear	70	907	0.0836	0.077	4.69	4.4
Workplace	0	Hosp./Sch.	3	1,828	0.0016	0.002	1	1
	1	Service	20	2,398	0.0084	0.008	5.12	5.08
	2	Indoor Office	22	2,176	0.0102	0.01	6.21	6.16
	3	Unemployed	116	1,730	0.0719	0.067	43.7	40.9
	4	Unclear	93	1,558	0.0635	0.06	38.6	36.4
Comorbidity	0	No	164	8,716	0.0192	0.019	1	1
	1	With	90	974	0.1018	0.092	5.31	4.91

**Figure 3:**
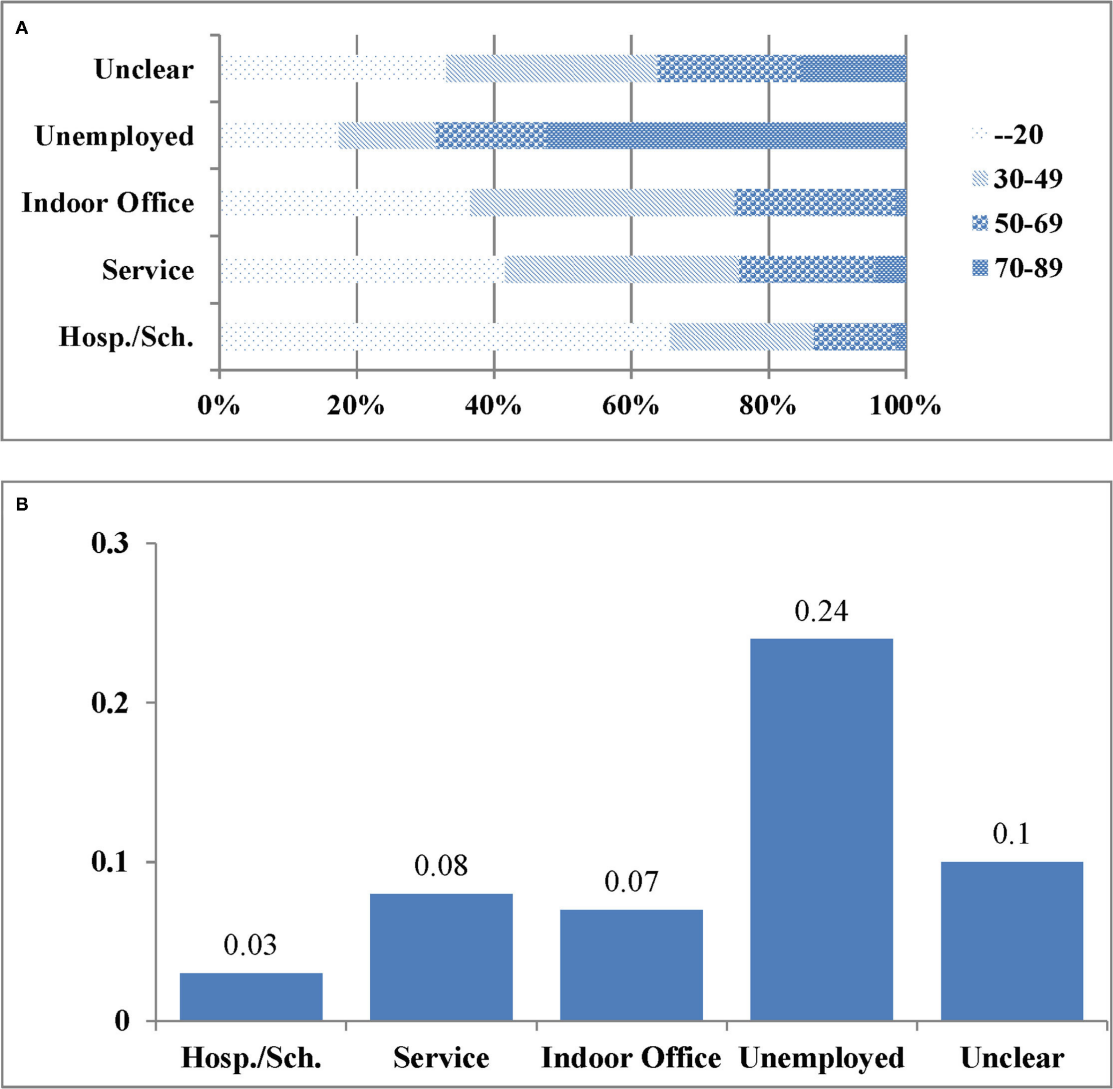
**(A)** The distributions of age by workplace. **(B)** The proportion of comorbidity by workplace.

Figure 3A displays large imbalances in age among workplaces. On average, hospital/school is the youngest, service is similar to indoor office; unclear is older than indoor office and unemployed the oldest. Figure 3B shows the proportion of comorbidity by workplace: unemployed is by far the highest and hospital/school the lowest, while service, indoor office, and unclear are similar to each other. These figures indicate that the OR and RR in Table 5 are misleading, since they are obtained ignoring the biases due to those imbalances in age and comorbidity. Hereafter, they will be termed as naïve OR and RR.

Table 6 obtained from stratifying by age shows the risks for the workplaces by age. Risks for unemployed and unclear are drastically reduced to 3.51 and 9.34 and 3.07 and 6.9 for age 50–69 and 70–99 years, respectively. The risks though may still be biased due to imbalances in sex, family status, and comorbidity. Since the stratifying method is not applicable to adjust for those confounders simultaneously, we had to resort to a logistic model. We estimate RR adjusted for those confounders by using a modified logistic model (27).

**Table 6:** The risk for workplace stratified by age.

**Age**	**Workplace**	**Freq**.	**Severe**	**Rate (%)**	**RR**
20: 0–29	0: Hosp./Sch.	1,202	0	0	–
	1: Service	998	0	0	–
	2: Indoor Office	796	0	0	–
	3: Unemployed	301	0	0	–
	4: Unclear	514	0	0	–
40: 30–49	0: Hosp./Sch.	382	0	0	–
	1: Service	818	2	0.24	1
	2: Indoor Office	837	5	0.6	2.5
	3: Unemployed	245	0	0	0
	4: Unclear	481	2	0.42	1.75
60:50–69	0: Hosp./Sch.	218	2	0.92	1
	1: Service	476	9	1.89	2.05
	2: Indoor Office	510	13	2.55	2.77
	3: Unemployed	279	9	3.23	3.51
	4: Unclear	326	28	8.59	9.34
80: 70–99	0: Hosp./Sch.	26	1	3.85	1
	1: Service	106	9	8.49	2.21
	2: Indoor Office	33	4	12.12	3.15
	3: Unemployed	905	107	11.82	3.07
	4: Unclear	237	63	26.58	6.9

### Naive OR and RR for Workplace by Using a Logistic Model

First, an ordinary logistic model with one covariate, workplace, is applied. The OR obtained from the model correspond to the naïve OR in Table 5. “Logistic for Odds” in Table 7 shows the results where OR = exp (Estimate). The ORs in Table 7 coincide well with the corresponding ORs in Table 5. A modified logistic model (28) resulted in “Logistic for RR” in Table 7, where RR = exp (Estimate). The naïve RR in Table 5 coincides exactly with the RR in Table 7, indicating a high accuracy of the estimates obtained from the modified logistic model.

**Table 7:** OR and RR estimated by using logistic models.

**Workplace**		**Logistic model for Odds**			**Model for RR**	
**Level**		**Estimate**	***P*-value**	**OR**	**Estimate**	**RR**
Hosp./Sch.	–	–	–	1	–	1
Service	Job*1	1.63	0.0085	5.11	1.626	5.08
Indoor Office	Job*2	1.83	0.003	6.22	1.818	6.16
Unemployed	Job*3	3.78	<0.0001	43.7	3.71	40.9
Unclear	Job*4	3.65	<0.0001	38.6	3.594	36.4

### RR Adjusted for Confounders

First, we applied an ordinary stepwise logistic model by using all the variables and stage as covariates to determine a best fit model. The selected variables are shown in “Logistic for Odds” in Table 8, where OR = exp (Estimate). The term age_60 × comorbidity is significant, indicating an age-dependent effect of comorbidity. Then, the modified logistic model (28), by using the selected variables as covariates, was applied. The results are shown in “Logistic for RR” in Table 8 where RR = exp (Estimate). RR for age is calculated by using the estimates exp (0·257 age_50–0·211 age_60) and that for comorbidity is exp (0·874 comorbidity−0·033 age_60 × comorbbidity).

**Table 8:** Results from ordinary stepwise and modified logistic models.

	**Logistic for Odds**						**Logistic for RR**	
**Variable**	**Estimate**	**95% CI**	**OR**	**95% CI**	**Estimate**	**RR**
Stage 0	−1.24	−1.54	−0.94	0.29	0.21	0.39	−1.059	0.35
Age_50	0.273	0.22	0.32					
Age_60	−0.218	−0.28	−0.16					
Sex	0.878	0.57	1.18	2.41	1.77	3.25	0.778	2.18
Family_1	0.488	0.31	0.67	1.63	1.36	1.95	0.396	1.49
Service	0.896	−0.34	2.14	2.45	0.71	8.5	0.928	2.53
Office/Gov.	1.133	−0.1	2.37	3.11	0.9	10.7	1.192	3.29
Unemployed	1.265	0.07	2.46	3.54	1.07	11.7	1.351	3.86
Unclear	1.886	0.7	3.07	6.59	2.01	21.5	1.779	5.92
Comorbidity	0.935	0.45	1.42					
Age_60 × Comorb	−0.035	−0.07	0					

**Figure 4:**
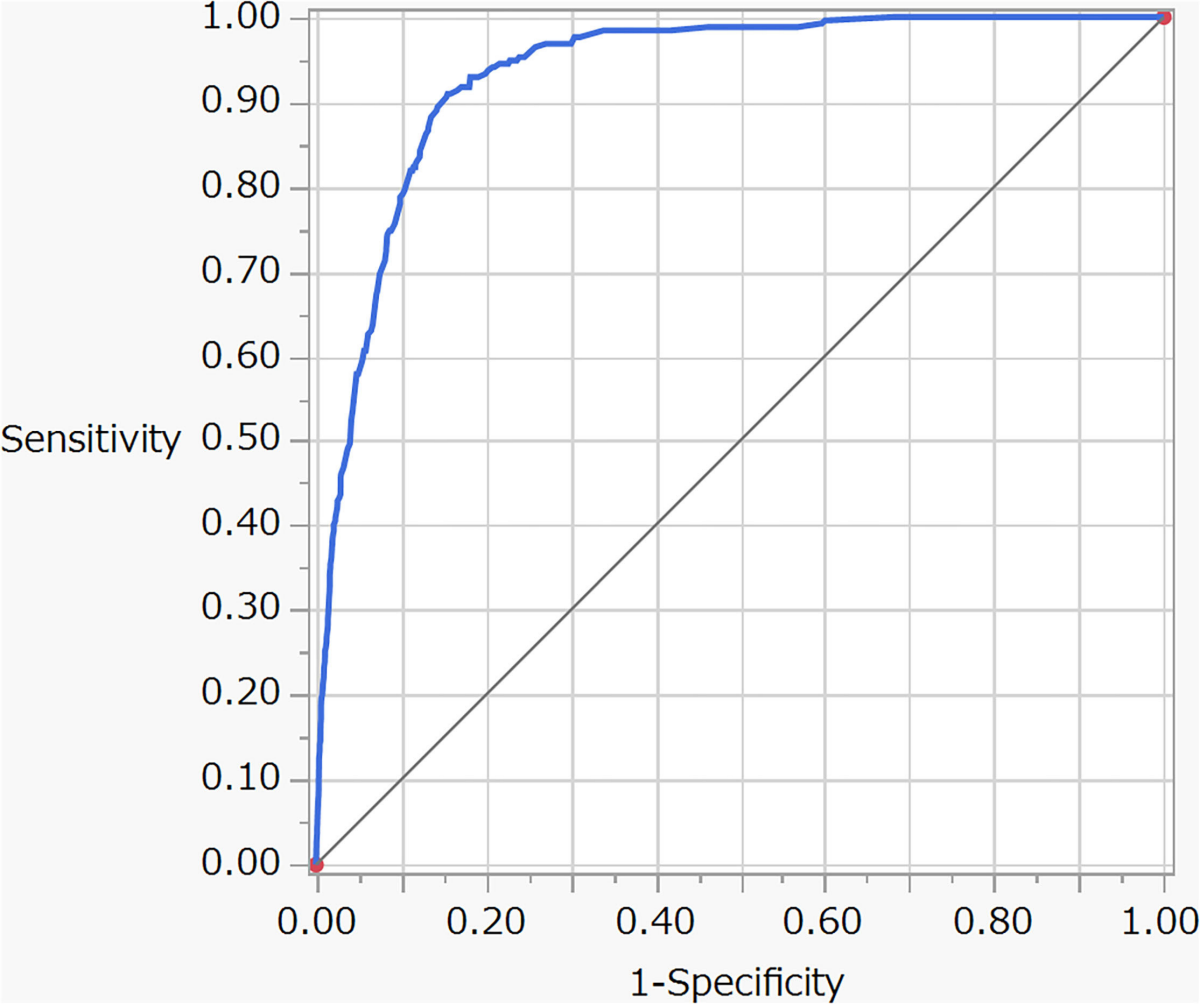
Receiver operating characteristic (ROC) curve for the logistic model described in Table 8.

**Figure 5:**
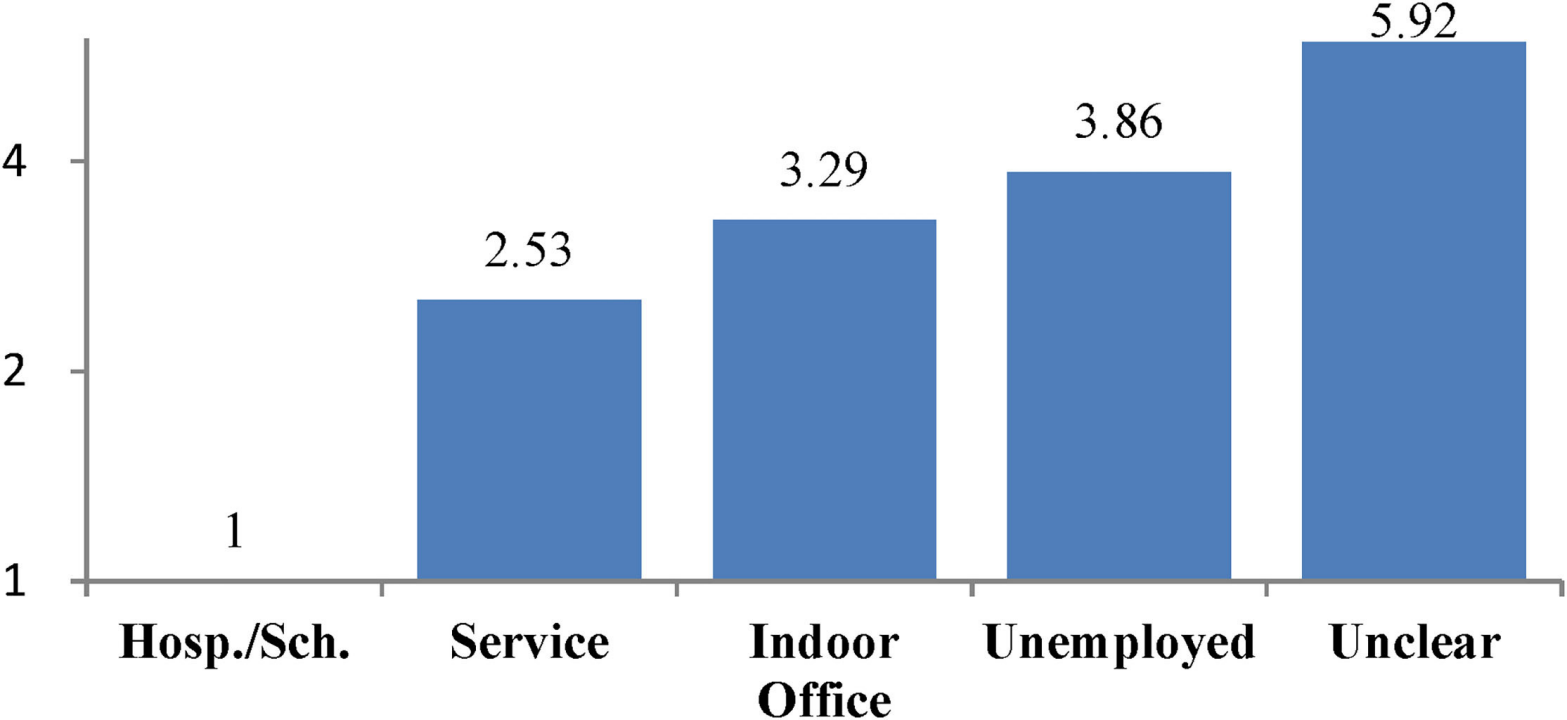
Adjusted relative risk (RR) for workplace.

The estimates for service and indoor office are not significant due to low frequencies of severe cases. The risk for each case is calculated from the estimates and the levels of the case according to the model equation. A receiver operating characteristic (ROC) curve obtained from the model shows a sensitivity of 0.85 and specificity of 0.9 (Figure 4), indicating a high discrimination ability between the severe and non-severe cases. The adjusted RRs for the workplaces are illustrated in Figure 5. Since the adjusted RRs still vary considerably, there should be unobserved, substantial risk factors intrinsic to workplaces.

## Discussion

### Prioritizing Cases According to the Risk of Disease Severity

The rapidly rising number of the COVID-19 cases in Japan and around the world has led to a shortage of ICU beds, medical staff, and the need to build emergency field hospitals (29). This shortage of ICU beds to treat the severe COVID-19 cases required the prioritization of patients to be hospitalized in ICU (30). The model described in Table 8 was obtained by using the ideal cohort data free from follow-up and selection biases and the ROC curve (Figure 4) demonstrates the high accuracy of the model to predict severe cases. Hence, the model could help to determine the COVID-19 cases at high risk of becoming severe at the initial diagnosis.

### Adjusted RR for Workplace

The large variations in the adjusted RR across the workplaces (Figure 5) suggest the existence of substantial, unobserved risk factors intrinsic to workplaces. Here, we consider two possible factors APHG and FDCE in the following discussion.

Adherence to public health guidelines (31) reduced hospitalization by 88% and mortality by 100% in Delaware (32). Since January 2020 in Japan, over 2,000 nurses and doctors of the self-defense forces (33) have supported healthcare efforts for patients with the COVID-19, yet none of them have been infected with the COVID-19. This demonstrates that the COVID-19 infections could be completely prevented or at least greatly reduced by strict APHG. With respect to the association between APHG and the severity of disease, Gandhi and Rutherford (23) argued that facial masking could become a form of “variolation” that may reduce the severity of disease among people who do become infected. As described in the introduction, their theory implies “following the appropriate public health guidelines might help to reduce the severity of disease among the infected cases.” The “variolation hypothesis” well explains the RRs for workplaces in Figure 5.

The risk of severe cases increases in the following order: hospital/school, service, indoor office, unemployed, and unclear. The lowest risk is hospital/school, despite high FDCE. In hospitals, healthcare professions, by virtue of their responsibilities to prevent nosocomial infections, demonstrate the public health guidelines for hospital staff and patients to follow. Schools are similar to hospitals in that faculty and staff set an example for observing health regulations to prevent interstudent transmission for students to follow. The results suggest that school is the safest place for students.

According to the data for the infection status of students summarized by the national government (Supplementary Material 3), school is the lowest risk place (5–7%) for primary and junior high students as compared to home (78–64%) and the other or unclear places (18–28%). The facts suggest that school is the safest place and students would visit higher risk places if schools were closed. This is in line with the above interpretation of the risks based on the variolation hypothesis.

The second lowest risk is, again despite high FDCE, the service industry. Service is not statistically significant (Table 8), since the frequency of severe cases is too low. These data do appear to provide evidence of high APHG usage within the service industry. Incentives for the high APHG in service include: professional courtesy to customers, deep-seated social customs, and the fact that infection would require quarantine or hospitalization without pay.

The risk in service industry is lower than that of indoor office. Indoor office employees have similar incentives to service industry regarding APHG, but with the addition that they are allowed to take paid leave. This paid leave system might have resulted in a lower APHG for indoor office, which, in turn, resulted in a higher risk than service industry. All the Japanese citizens receive benefits from the universal health insurance system regardless of income and income disparities are not very large among regular employees in Japan. Hence, differences in APHG should have a greater impact on risk than income disparity among regular employees. This might explain why service carries a lower risk than indoor office despite service having lower incomes on average than indoor office.

High FDCE should normally result in a potentially higher risk of infection; in fact, a high FDCE associated with a high risk of infection was observed in a cross-sectional study on 104 retail workers in Massachusetts (34). According to the “variolation” hypothesis (23), however, a high FDCE combined with a high APHG, such as in the service industry, should result in generating immunity and helping to reduce the severity of disease among people who do become infected, which it does in our case. This hypothesis may also apply to the low risk in hospital/school.

“Unemployed” has a higher risk than indoor office. This could be because those in unemployed may be less motivated to follow APHG than indoor office. Research to ensure exact reasons and to promote effective measures among any high-risk group is urgent for reducing severe future infections.

Unclear shows the highest severity risk. To the best of our knowledge, no studies have investigated the risk of the severity of the COVID-19 for the unclear category. Normally, requesting occupation when recruiting volunteer participants can result in eliminating or ignoring this category (20, 21), making it generally difficult to study its characteristics. In this study, however, data on the cases of unclear were collected in the same way as the other occupations, since it was obligatory by law.

While age, sex, family status, and comorbidity are confounders that should be adjusted for estimating the risk, income is a so-called “intermediate factor” in epidemiology that should not be adjusted in eliminating risk. Professions affect income and, in turn, income affects the risk. The higher risk for unemployed and unclear might be partly due to low APHG caused by low income. Nevertheless, we should consider effective measures to promote APHG for unemployed and unclear because low income is intrinsically associated with unemployed and unclear.

The results of this study suggest that promoting APHG would be the most effective measure in preventing the disease severity irrespective of FDCE. Therefore, we conclude that measures for promoting APHG should be coupled with lockdowns to mitigate both the disease severity and the negative impact of lockdowns. In addition, since the food industry seems to be taking effective measures to promote APHG, it should not be targeted indiscriminately by lockdowns. Rather, it would be more effective to help reinforce their efforts to prevent infection; for example, by installing CO_2_ concentration measuring devices (35) for assessing their efforts to prevent droplets infections or by providing financial reinforcementso that they can advance their own measures for prompting customers to follow APHG. In addition, the higher likelihood that lower ventilation rates are associated with higher infection risks (36) could help explain higher risk for indoor office than service, since many high-rise offices cannot open windows and the air is only filtered for quality and not pathogens; therefore, providing adequate ventilation would be in many cases a low technology, low cost way to reduce transmission rates. The evidence-based workplace-specific measures should be tested and corroborated in order to effectively control the infection with fewer negative effects.

### Study Limitations

Our data is not subjected to the biases that are usually associated with cohort studies such as follow-up biases, selection biases, and biases due to misclassifications in outcome or covariates. However, since our data were published by the Osaka Prefecture, which strictly follows the common privacy rules, there are limitations in available information. To gather information on the background of individuals in unclear, we must submit a study protocol based on the results of this study to the Institutional Review Board. If approved, the Osaka Prefecture government will help us conduct the study.

Frequency of direct customer exposure and APHG are assumed to be the major variables causing the large differences in the workplace-specific risks. FDCE for a workplace is evaluated based on the subjective observations. It is a study limitation for FDCE to be currently assessed subjectively, not objectively. For example, the questionnaire used by Lan et al. to evaluate FDCE of employees is also based on the subjective evaluation of the employees. Nevertheless, we feel that FDCE is a key concept to understand the relationship between the risk and workplaces. Mission of epidemiology is to discover effective countermeasures based on uncertain observational information. We hope that this research will recognize the importance of FDCE and devise future objective measurement methods.

While the workplace-specific severe disease risks that we have obtained might not be in line with the beliefs of some policymaking, lockdown planners, these risks are facts obtained by applying standard statistical methods to cohort data of the PCR-positive COVID-19 infected cases of Osaka, which were, without exception, confirmed as either recovered or dead on the day of discharge.

## Data Availability Statement

The original contributions presented in the study are included in the article/Supplementary Material, further inquiries can be directed to the corresponding author/s.

## Author Contributions

YN and TN designed the cohort study. HM organized a team for collecting data for analysis as described in Supplementary Material. TN and HM performed statistical analysis. TN and TS wrote the draft and HM elaborated tables and figures. HC reviewed literature and discussed the results from a medical point of view. All authors approved the final manuscript for submission.

## Conflict of Interest

The authors declare that the research was conducted in the absence of any commercial or financial relationships that could be construed as a potential conflict of interest.

## Publisher's Note

All claims expressed in this article are solely those of the authors and do not necessarily represent those of their affiliated organizations, or those of the publisher, the editors and the reviewers. Any product that may be evaluated in this article, or claim that may be made by its manufacturer, is not guaranteed or endorsed by the publisher.
