# Supplementary material for: Impact of Workplace on the Risk of Severe COVID-19
Source: Front Public Health. 2022 Jan 5;9:731239. doi: 10.3389/fpubh.2021.731239 (PMC8766507; doi:10.3389/fpubh.2021.731239)
Supplement: Supplementary file 1 [file Data_Sheet_1.pdf]

Supplementary Material

1. Notification of Outbreak Form

Table displays the Notification of Outbreak Form translated from Japanese.

Table: Notification of Outbreak Form

		**Report of COVID-19 Case Form**					
				**Date of Report: :**		**Year Month Day**	
	**Name**						
**Doctor**							
**Hospital**							
**Address**							
**Phone**							
**1 Type of Case (Cadaver) Diagnosed**							
**Case (Confirmed)**		**Asymptomatic ・ Pseudo Symptom ・ Case Cadaver ・Pseudo Symptom Cadaver**					
		**Do not report Psudo Symptom case not to be hospitalized**					
**2 Name**		**3 Sex**	**4 Date of Birth**			**5 Age at Diagnosis**	**6 Profession**
		**M F**	**Year**	**Month**	**Day**	**Year Month**	
**7 Address**							**Tel**
**8 Address of Work**							**Tel**
**9 Name of Parents**		**10 Address of Parents**				**9 and 10 are for Minor**	
							**Tel**
**11 Symptom**					**18 Cause, Route, Area of Infection**		
**12 Method of Diagnosis**					**19 Measure for treatment or prevention of infection**		
			**Yesr**	**Month**	**Day**		
**13 Date of First Diagnosis**							
**14 Date of Diagnosis**							
**15 Date of Infection(Estimate)**							
**16 Date of Onset**							
**17 Date of Death**							
					**Currently Hospitalized?:**		
					**Comorbidity ?**		
					**Immune Depression?**		
					**Pregnant ?**		
					**Severity :**		
					**Need Hospitalization ?**		
					**Need Telemedicine ?**		

Persons with unknown occupations are those who failed to answer question 6 “Profession” in interviews conducted by doctors and public health professions. This medical form is for internal use and private information such as name and address are not available to the public.

2. Method for access to the original data and transformation to EXCEL file

This section describes how to access to the original data and transform it to an EXCEL file.

1. First we accessed Osaka prefecture's COVID19 data site:
http://www.pref.osaka.lg.jp/hodo/index.php?site=fumin&k_flg=1

2. Enter the period from "2021/04/09" to "2021/04/16" and type "新型コロナウイルス感染症患者の発生"、or "Outbreak of patients with new coronavirus infection" in the keyword window, and click "検索"、or "Search". A list of titles will appear.

3. Click a title to move to the explanation of the corresponding cases. Click "Pdf file" to obtain the Pdf file of the cases. Here is an example of the results:
http://www.pref.osaka.lg.jp/hodo/index.php?site=fumin&pageId=39760

4. Copy Pdf file. Paste it to a Text file, and Open it by EXCEL specifying blank as a delimiter. This creates an EXCEL file for the data of the cases.

5. Comorbidities, if any, are described in Notices column.

3. Infection status of children and students reported in 2020/06/01-- 2021/04/15

by Ministry of Education

Affiliation	Numbers			Infection Route							
	Total	Symptomatic		At Home		At School		Other		Unclear	
**Primary**	6183	2153	35%	4817	78%	282	5%	410	7%	661	11%
**Junior High**	4072	2098	52%	2619	64%	291	7%	266	6%	882	22%
**High**	7046	4420	63%	2393	34%	1704	24%	575	8%	2352	33%
**Special**	269	107	40%	135	50%	23	9%	60	22%	51	19%
**Total**	17570	8778	50%	9964	57%	2300	13%	1311	7%	3946	22%

Cited from "Severe acute respiratory syndrome in school Hygiene management manual (2021.4.28 Ver.6)", p. 5 "Infection status of children and students"

4. Brief Description of the Promotion of Infection Prevention Measures of Osaka Prefecture during the Study Period

From April 7 to May 6, 2020: *Emergency Measures of Osaka Prefecture*

●**Request for staying at home**

Osaka residents are requested to stay at home, especially requested to refrain from going out to downtowns at night time, where "Three Cs" (*Closed spaces, Crowded places and Close-contact settings*) overlap.

Necessary activities for daily lives such as going to hospital, shopping for foods and commuting are exception.

From April 14 to May 6, 2020

●**Request for Facility Use Restriction**

1 **Facilities NOT requested to close : Facilities essential for social lives, Social welfare facilities, etc.**
⇒**Requested to take appropriate infection prevention measures.**

2 **Facilities requested to close: Entertainment, exhibition, sports, and education facilities**
⇒**If they don't obey: Facilities' names will be publicized**

3 **Facilities requested use restrictions (The following facilities with floor areas over 1000m^2)**
Universities, tutoring schools, museums, hotels, inns, and commercial facilities
⇒**If they don't obey: Facilities' names will be publicized**

From April 25 to May 6, 2020: *Request During Golden Week Holidays*

●**Request for Cooperation at Supermarkets, etc.**

1 **Set up the priority time zone for expectant mothers, senior citizens, persons with disabilities, and persons with "help mark" badges.**
2 **Indicate the positions to line up for each cashier**
3 **Suspend specific day and time services such as discount sale and bonus-point campaigns as much as possible.**
4 **Control the entry of shoppers when approx. 2m social distance can't be kept.**

From May 16 to 31, 2020: *Emergency Measures after May 16*

●**Request for staying at home**

Reduce social contact by at least 70%, hopefully 80%. Above all, the following is strongly requested.

1 **Refraining from travels over prefectures, such as nonessential homecoming visits or trips.**
2 **Refraining from going out to downtown at night, such as going to eateries with hospitality services.**
3 **Thoroughly avoiding "*Three Cs", as well as practicing "New Lifestyle" to prevent infections from spreading.(e.g. Teleworking).**

From April 25 to May 6, 2020: *Request During Golden Week Holidays*

●**Request for Cooperation at Supermarkets, etc.**

1 **Set up the priority time zone for expectant mothers, senior citizens, persons with disabilities, and persons with "help mark" badges.**
2 **Idicate the positions to line up for each cashier**
3 **Suspend specific day and time services such as discount saleand bonus-point campaigns as much as possible.**
4 **Control the entry of shoppers when approx. 2m social distance can't be kept.**

__From May 16 to 31, 2020: *Emergency Measures after May 16*__

●Request for staying at home

Reduce social contact by at least 70%, hopefully 80%. Above all, the following is strongly requested.

1 Refraining from travels over prefectures, such as nonessential homecoming visits or trips.

2 Refraining from going out to downtown at night, such as going to eateries with hospitality services.

3 Thoroughly avoiding "*Three Cs", as well as practicing "New Lifestyle" to prevent infections from spreading. (e.g. Teleworking).

__From 7.16 --: Promotion of Infection Prevention Measures in *Minami area (one of the major nightlife areas*）__

1 Visiting eateries with entertainment services individually to request them to place a "declaration of infection prevention sticker" and to take other infection prevention-related measures.

2 When infections occur in a facility where prevention measures are not taken, the facility's name is to be publicized based on the Infection-related law.

3 Ventilation should be thoroughly conducted with on-site inspections in bars, night clubs, eateries with entertainment services.

__From August 6 to August 20, 2020: *Requests at the Yellow Stage (Warning)*__

When either of the following criteria is met, we move on to the Yellow Stage2

① Beds for severe symptom patients：Approx. 35% or Beds for slight/mild symptom patients: Approx. 50%

② When measures such as facility use restriction, etc. are taken.

Additional requests to Facilities in __*Minami area (one of the major nightlife areas*）__

Facilities in Minami area		Measures
Eateries with entertainment services (cabarets, host clubs, etc.) Eateries providing alcohol（bars, night clubs) and karaoke parlors	Facilities that don't comply with the guidelines (or don't have a "declaration of infection prevention sticker."）	Request business closure
	Facilities that comply with the guidelines noted above (or have stickers)	Request reduction of business hours（5:00pm to 8:00pm）
Other eateries that provide alcohol (pubs, etc.)		Request reduction of business hours（5:00pm to 8:00pm）

●*Requests to Osaka residents.*

・Avoid the Three Cs (Closed spaces, Crowded places and Close-contact settings)

・Refrain from visiting nightlife facilities unless they thoroughly comply with the guidelines

・To senior citizens and people with underlying diseases and their families: Refrain from visiting facilities mentioned above.

●***Requests to economic communities and universities.***

・Refrain from a (drinking) party of five or more people
・Thoroughly comply with guidelines determined for each industry and university.
・Encourage to raise the ratio of teleworking up to 70%
・Let people who are in bad health stay home. Receive a PCR test even with slight symptoms
・Use the facilities that have a "declaration of infection prevention sticker"

Appropriate Preventive Measures for Infections

Aim	Examples
Prevent people with fever from entering	・Taking employees' temperature and checking their health, and suspend them if they have fever of 37.5C or higher and/or poor physical condition
	・Taking guests' temperature and checking their health and stop them from entering if they have fever of 37.5℃ or higher and/or poor physical condition
Prevent Three Cs (Closed spaces, Crowded places and Close-contact settings)	・Limiting entry, taking measures to avoid letting guests in lines closely
	・Air ventilation (opening windows on two different directions at the same time)
	・Cancelation of meeting where attendees get close(telephone or web meeting is recommended)
	・Changing layout of office to secure enough space, etc.
Prevent infection by contact or droplet transmission	・Encouraging employees to wear a face mask, wash and disinfect hands and cough etiquette
	・Encouraging guests to wash and disinfect hands and implement cough etiquette
	・Disinfecting offices and shops regularly
	・Dividing space and other measures at counter service
Prevent infection during the operating time	・Encouraging staggered working hours, commuting by car, etc.
	・Limiting the number of employees at the office(home teleworking is recommended)
	・Cancelation of official trips (telephone or web meeting is recommended)

4. Data Availability Statement

EXCEL dataset for this study will be uploaded as Supplementary Material on acceptance.
